# The source, fate and prospect of antibiotic resistance genes in soil: A review

**DOI:** 10.3389/fmicb.2022.976657

**Published:** 2022-09-23

**Authors:** Binghua Han, Li Ma, Qiaoling Yu, Jiawei Yang, Wanghong Su, Mian Gul Hilal, Xiaoshan Li, Shiheng Zhang, Huan Li

**Affiliations:** ^1^Institute of Occupational and Environmental Health, School of Public Health, Lanzhou University, Lanzhou, China; ^2^State Key Laboratory of Grassland Agro-Ecosystems, Center for Grassland Microbiome, Lanzhou University, Lanzhou, China; ^3^Chongqing Key Laboratory of Development and Utilization of Genuine Medicinal Materials in Three Gorges Reservoir Area, Faculty of Basic Medical Sciences, Chongqing Three Gorges Medical College, Wanzhou, China

**Keywords:** antibiotic resistance genes, resistomes, microbiome, horizontal gene transfer, heavy metals

## Abstract

Antibiotic resistance genes (ARGs), environmental pollutants of emerging concern, have posed a potential threat to the public health. Soil is one of the huge reservoirs and propagation hotspot of ARGs. To alleviate the potential risk of ARGs, it is necessary to figure out the source and fate of ARGs in the soil. This paper mainly reviewed recent studies on the association of ARGs with the microbiome and the transmission mechanism of ARGs in soil. The compositions and abundance of ARGs can be changed by modulating microbiome, soil physicochemical properties, such as pH and moisture. The relationships of ARGs with antibiotics, heavy metals, polycyclic aromatic hydrocarbons and pesticides were discussed in this review. Among the various factors mentioned above, microbial community structure, mobile genetic elements, pH and heavy metals have a relatively more important impact on ARGs profiles. Moreover, human health could be impacted by soil ARGs through plants and animals. Understanding the dynamic changes of ARGs with influencing factors promotes us to develop strategies for mitigating the occurrence and dissemination of ARGs to reduce health risks.

## Introduction

Antibiotic resistance refers to the mechanism in which microorganisms can resist a high concentration of antibiotics, while other organisms are susceptible to these antibiotics (Martinez et al., 2007). The sum of all ARGs in soil environment is called “soil resistome” (Gorovtsov et al., 2018). ARGs are considered as environmental pollutants of emerging concern (Tan et al., 2018), and its global spread and dissemination pose a great threat to human health. Human pathogens carrying various ARGs can generate superbugs, which persist after antibiotic treatment and eventually lead to human death. Antibiotic resistance is reported to cause 700,000 people deaths a year, more seriously, it is estimated to account for 10 million deaths by 2050 if uncontrolled (O’Neill, 2016).

Most antibiotics are natural compounds or originate from natural compounds, produced by different kinds of bacteria, fungi or plants (Davies and Davies, 2010). Before the introduction of synthetic antibiotics, the existence of natural antibiotics may exert continuous selective pressure on microorganisms, but most microorganisms were susceptible to antibiotics before the era of antibiotics discovery (Davis and Anandan, 1970; Hughes and Datta, 1983). From 2010 to 2030, it is estimated that the use of antibiotics in animal production worldwide will increase by 67%, and China is predicted to increase by 99% (Van Boeckel et al., 2015). The huge market demand for antibiotics and its inappropriate use globally have promoted the increase and spread of ARGs in soil microorganisms (Cheng et al., 2013; Gorecki et al., 2019).

Soil is one of the largest environmental reservoirs for ARGs (Forsberg et al., 2012), accounting for about 30% of the known ARGs, and it is also one of the most complex ecosystems in terms of niches and biodiversity. At present, several main antibiotics used by humans are screened and cultured from soil (30%), and more than 80% of antibiotics used clinically come from soil bacteria. ARGs are indigenous in soil (World Bank, 2017), which may resist to β-lactam, tetracycline and glycopeptide, and these ARGs are detected in 30,000-year-old Beringian permafrost sediments, and exogenous ARGs can also be introduced into the soil by applying organic manure, sewage discharge, and irrigation of wastewater treatment plants (WWTPs) in densely populated areas. Thus, soil can act as a reservoir and potential source of ARGs, which poses a threat to the environment and human health.

**Figure 1:**
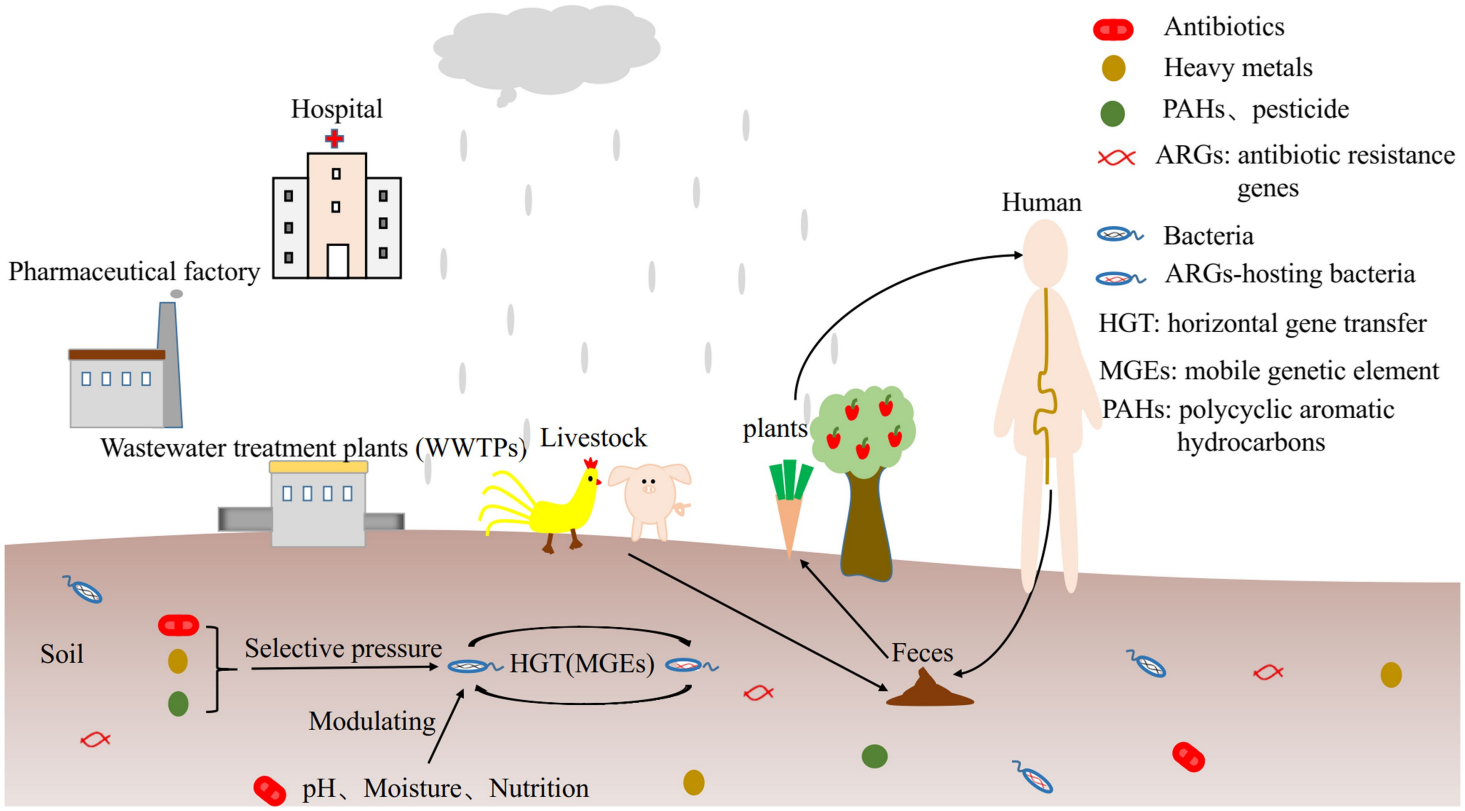
The influencing factors of ARGs.

The influencing factors on ARGs profiles are different depending on soil types. Microbial community structure, antibiotic type and concentration, soil physicochemical properties (such as pH, humidity, nutrition and temperature), heavy metals, polycyclic aromatic hydrocarbons (PAHs) and pesticides can exert selective pressure on the soil resistomes (Figure 1) and interact with each other. Given that the potential transmission of environmental ARGs to human commensals and pathogens, investigating the source and transmission mechanism of antibiotic resistance in soil, their relationships with microorganisms, mobile genetic elements (MGEs), soil physicochemical properties and various pollutants will contribute to managing antibiotic resistome in soil, and develop effective mitigation strategies to improve human health.

Despite these advances mentioned above, many knowledge gaps still remain in the field of soil antibiotic resistance, as described below. What inexpensive and easy-to-operate technologies can effectively treat emissions of ARGs-containing substances? What are the mechanisms that affect soil ARGs by soil special substrates, such as clay and humic acid? What are the complex interactions and molecular mechanisms between soil ARGs and environment and human health? What are the individual and compound effects of various environmental and biological factors on soil ARGs? More researches are needed to address these questions.

## Antibiotic resistance gene classes distribution across soil

Some ARGs are indigenous in soil, and may originate from soil indigenous bacteria. β-Lactam, tetracycline and vancomycin resistance genes can be detected in some relatively primitive environments with no or limited human impacts (Martinez et al., 2015), such as permafrost (Mindlin et al., 2008; Perron et al., 2015), Antarctic soil (Van Goethem et al., 2018) and Qinghai-Tibet Plateau (Yang et al., 2019; Li B. et al., 2020). Therefore, ARGs are natural and ancient, preceding the selective pressure exerted by man-made antibiotics. ARGs detected in the relatively primitive Qinghai-Tibet plateau soil with high altitude and limited human activities are mainly uncommon glycopeptides (such as vancomycin), rifamycin (Van Bambeke, 2004; Damodaran and Madhan, 2011) and a small part of β-Lactamase resistance genes (LRA-3, LRA-9 and LRA-5). Amycolatopsis in the soil can secrete vancomycin and rifamycin (McIntyre et al., 1996; Tan et al., 2006). Although β-Lactam antibiotics are widely used in clinical treatment, the three genotypes of LRA-3, LRA-9 and LRA-5 have not been reported in clinical pathogens (Li B. et al., 2020). Seven kinds of ARGs, including quinolones, aminoglycosides, β-Lactam, macrolide-lincomycin-streptomycin B (MLSB), multidrug, sulfonamides and tetracycline resistance genes, were detected in 24 pristine forest soils in China, in which aminoglycosides and quinolones resistance genes were dominant (Song et al., 2021). Thirty classes of ARGs resisting to the sulfonamides, tetracyclines, aminoglycosides, quinolones, macrolides and β-Lactam were detected in polar sediments. Among these ARGs, *sul* genes were the most common, and the abundance (10^−8^ ~ 10^−6^ copies/16S rRNA gene copies) was about 2 to 5 orders of magnitude lower than that other areas having higher anthropogenic activities (10^−4^ ~ 10^−2^ copies/16S rRNA gene copies; Tan et al., 2018). In the relatively primitive Qinghai-Tibet plateau wetlands, FCAs (fluoroquinolone, quinolone, florfenicol, chloramphenicol, and amphenicol resistance genes), β-Lactamase, MLSBs, aminoglycoside and tetracycline resistance genes were dominant (Yang et al., 2019). In conclusion, the types of dominant ARGs detected in different soil types are distinct (Table 1).

**Table 1:** Antibiotic resistance genes (ARGs) and mainly influencing factors in different soil types.

Soil type	ARG numbers	Dominant ARGs	ARG abundance	Mainly influencing factors	Reference
24 Pristine forest soils (broadleaf forests)	25 Subtypes of the 30 target ARGs	Aminoglycoside (36.3%), quinolone (27.7%), MLSB^a^ (17.5%) resistance genes	Average absolute abundance: 1.78 × 10^5^ copies/g soil Average relative abundance: 7.22 × 10^−3^ ARG copy numbers/16S rRNA gene copy numbers	Physicochemical factors (e.g., temperature, total phosphorus) (65.8%), microbiota (48.3%), spatial factors (longitude) (26.79%), and MGE (3.6%).	Song et al. (2021)
5 Forest soil from north to south (boreal forest, temperature mixed coniferous forest, temperate deciduous forest, subtropical (evergreen broadleaf) forest and tropical (rainforest) forest)	160 ARGs	Multidrug (22.4%), β-lactam (21.8%), aminoglycoside (13.5%), MLSB (14.1%), tetracycline (11.2%) and vancomycin (8.2%) resistance genes	–	MGEs, microbiota, herbs and pH	Hu H-W. et al. (2018)
Amazon rainforest soils	215 ARG subtypes	Multidrug resistance genes	0.243 Copies/16S rRNA gene	Bacterial community composition	Qian et al. (2021)
Deciduous forest	7 ARGs	Sul1, ermB, vanA, aph(3′)-IIa, aph(3′)-IIIa, tet(W) and blaTEM-1	–	–	Radu et al. (2021)
Deep forest in Yunnan	–	Multidrug, MLS (macB) resistance genes	–	–	Zheng et al. (2021)
Paddy soil in South China	16 ARGs, corresponding to 110 ARG subtypes	Multidrug (38–47.5%), acriflavine (16.4–21%), MLS (13.2–20.7%), bacitracin (5.4–12.5%) resistance genes	7–10 ppm	Microbial communities, pH	Xiao et al. (2016)
Paddy soil in Shaoxing City	5 ARGs	TetB, tetC, tetW, sul1, sul2	2.37 ~ 5.01 log10-transformed copies/g dry weight	Microbial community	Lin et al. (2016)
Paddy soil in the Lake Tai Basin	>6 ARGs	Multidrug (>90%) resistance genes	–	MGEs	Zhang et al. (2021)
Paddy soil in Hunan province	119 ARGs	Multidrug (17.6%), tetracycline (16.8%), aminoglycoside (16.0%), MLSB (15.1%) and β lactam (14.3%) resistance genes	10^9^ ~ 1.2 × 10^12^copies/g dry soil	Bacteria, MGEs, As	Zhao et al. (2020)
Urban soil in Belfast, Northern Ireland	164 ARGs	β-lactams (23%) and multidrug (23%) resistance genes	6.8 × 10^2^ ~ 1.7 × 10^8^ copies/g soil	MGEs, heavy metals (Cu, Zn, etc), pH	Zhao Y. et al. (2019)
Urban soil in Victoria, Australia	40 ARGs	β-lactam (>23%), MLSB (16.34%), and quinolones and fluoroquinolones (11.76%) resistance genes	–	Reclaimed water irrigation, MGEs, bacterial community composition, pH, total nitrogen	Han et al. (2016)
Urban soil in Greater Melbourne, Australia	217 ARGs	Multidrug (52.22%), MLSB (18.50%) and β-lactamase (12.30%) resistance genes	Around 10^−3^ copies/16S rRNA gene	MGEs, industrial distribution	Yan et al. (2019)

a Macrolide-lincosamide-streptogramin B.

tet, tetracycline resistance genes; sul, sulfonamide resistance genes; erm, macrolide resistance genes; bla, β-lactam resistance genes; van, vancomycin resistance genes; aph (3′)-IIa, aminoglycoside resistance genes.

Tetracycline resistance genes are generally dominant in various soil types, such as agricultural soil (Durso et al., 2012; Cadena et al., 2018), and ~ 60% of soil-derived strains are resistant to tetracycline (D’Costa et al., 2006). The possible reason is that tetracycline is widely used in animal husbandry and is one of the top five antibiotics most commonly used in China (Zhang et al., 2015). Tetracycline, an indigenous and ancient antibiotic, exerts strong selection pressure on ARGs, which have been detected in Beringian permafrost formed 30,000 years ago (D’Costa et al., 2011) and polar sediments (Tan et al., 2018).

Vancomycin resistance genes are widely distributed and detected in most wetlands of relatively primitive Qinghai-Tibet Plateau (Yang et al., 2019) and other types of soils (D’Costa et al., 2011), which pose a great threat to public health because vancomycin is the last resort against Gram-positive bacteria (some strains are resistant to most antibiotics; Hubbard and Walsh, 2003).

Multidrug resistance genes, one of the dominant ARGs in soil, explain the emergence of multidrug-resistant bacteria in the clinic. Several common ARGs have been detected in the river beach soils of Port Philip Bay and Yarra River in Victoria, Australia, among which multidrug resistance genes were the most abundant, accounting for 32.7% of the total number of detected ARGs (Zhang Y. et al., 2019).

## The origin of antibiotic resistance genes

### Natural antibiotic resistance genes in soil

Microorganisms in soil secrete some antibiotics to compete for resources (high concentration antibiotics; Newman et al., 2003) or communicate (low concentration antibiotics; Linares et al., 2006). Microorganisms resist antibiotics to protect themselves from “suicide” (i.e., the “producer hypothesis”; Cundliffe, 1989; Wright, 2007; Davies and Davies, 2010; Cordero et al., 2012). Microorganisms without resistance can be killed or inhibited by antibiotics. Therefore, according to Darwin’s “arms-shields race” hypothesis, in order not to be killed or inhibited by antibiotics, microorganisms adjacent to antibiotics producers acquire antibiotic resistance through gene mutation and expression of latent genes under the selective pressure of antibiotics (Cytryn, 2013). Therefore, some ARGs are indigenous in the soil itself. And it is estimated that the origin of natural antibiotics is before 2 Gyr ~ 40 Myr, thereby the antibiotic resistance should also be equally ancient (Hall and Barlow, 2004).

### Antibiotic resistance genes with composting

In addition to the indigenous resistome, resistome obtained through various ways are called the “acquired resistome.” As common drugs and feed additives, antibiotics are widely used and even abused to treat diseases and promote animal growth. Even after taking antibiotics for a long time, antibiotic-resistant bacteria (ARB) can continue to exist in the intestine for many years (Clemente et al., 2015). Up to 30 ~ 90% of antibiotics are not fully absorbed or metabolized after ingestion and excreted through faeces in the form of original drugs, conjugates and by-products (Johnson et al., 2016; Qian et al., 2018). After animal manure is applied to soil, the residual antibiotics exert selective pressure on microorganisms, then conferring antibiotic resistance (Guo et al., 2018). A large number of ARGs were detected in human faeces and animal intestines and faeces. For the sake of amending soil and increasing soil nitrogen source and organic matter (Garcia-Pausas et al., 2017), applying manure has become a routine operation. However, this operation introduces fecal ARGs into the soil (Graham et al., 2016; Peng et al., 2017). It is reported that there were no significant differences in ARGs abundance and types between soil and animal faeces in a relatively primitive Tibetan environment (Chen B. et al., 2016). Long term application of livestock manure can increase the diversity and abundance of ARGs (Li S. et al., 2020). The number of observed ARGs in manured soil increased significantly and continuously during the three consecutive years of fertilization from 2015 to 2017 compared with the treatment without manure application (fertilized pig manure 68–92, fertilized chicken manure 70–84, fertilized cow manure 69–83; Liu et al., 2021). However, other researchers have pointed out that long-term application of organic fertilizer does not always increase the abundance of ARGs. Firstly, the composting process and high temperature (the temperature above 70°C can completely and directly degrade bacterial DNA) can reduce the abundance of ARGs by killing the bacterial hosts (Guo et al., 2019). The total abundance of ARGs decreased by 90.19–91.87% on the 17th day of composting (Guo et al., 2019). Secondly, the exogenous ARG-containing bacteria cannot acclimatize to soil environment and can be inhibited by soil native bacteria; thus, some faecal ARG-containing bacteria can only survive temporally in the soil. After the application of manure, the abundance of ARGs may subsequently decreased (Sun et al., 2015c). The total relative abundance of ARGs decreased gradually with composting, from 2,910 ppm at day 0 to 1,200 ppm and 700 ppm in day-71 and day-171 composting, respectively (Zhang et al., 2020). In addition to manure, the decomposition of animal carcasses, especially fish, livestock and poultry, will also introduce ARGs into the soil. For example, the absolute and relative abundance of total ARGs were enriched 536.96 and 18.16 folds in the Crucian carps carcass soil, respectively (Feng et al., 2021).

### Antibiotic resistance genes in WWTPs

As the interface between humans and the environment, WWTPs in densely populated cities contain various antibiotics, ARBs and ARGs discharged from farms, hospitals and pharmaceutical industries. Microorganisms are subjected to multiple selection pressures in WWTPs, making it possible for horizontal gene transfer (HGT) between environmental bacteria and pathogenic bacteria (Rizzo et al., 2013), and microbial opportunists such as multidrug-resistant bacteria occur after exposure to various toxic compounds (Tello et al., 2012). The WWTPs aim to remove carbonaceous materials, nutrients, and pathogenic bacteria, and are not explicitly used to remove antibiotics and ARGs. The wastewater discharged from the WWTPs introduces and enriches many antibiotics and ARGs, which may pollute soil. High concentration of sulfamethoxazole (18 μg kg^−1^) was detected in the soil adjacent to WWTP of Puchukollo, while sulfamethoxazole has not been observed in other soil sampling sites staying away from WWTP (Archundia et al., 2017). Therefore, WWTPs can act as a source of pollutants (Guo J. et al., 2017), which contains plenty of resistance genes.

## The impacting factors of antibiotic resistance genes

### The association between antibiotic resistance genes and microorganisms

Soil microorganisms are main producers of antibiotics and their derivatives, which can equip themself to compete for limited resources. Actinomycetes are well-known bacteria that produce antibiotics (Lima-Mendez et al., 2015; Zhang et al., 2020; Song et al., 2021). Approximately 5–6% of soil bacteria are actinomycetes, most of which can secrete tetracycline. Soil microorganisms are also potential hosts of ARGs. In the study of soil metagenomics, Proteobacteria and Actinobacteria are the most common predictive hosts of multidrug resistance genes (D’Costa et al., 2006; Rafiq et al., 2017; Guo et al., 2019; Yang et al., 2019). All isolates carrying gram-negative *sulI*/*sulII* genes belong to Proteobacteria (Razavi et al., 2017).

Previous studies have shown that bacterial community structure mainly determines the soil ARGs profiles (Forsberg et al., 2014), and bacterial phylogenetic and taxonomic structure significantly affect resistant components in the soil (Chen Q. et al., 2016). Therefore, ARGs profiles can be affected by changing bacterial communities (Xie et al., 2018). Microbial diversity is negatively linked to the abundance of resistance genes (Van Goethem et al., 2018; Chen et al., 2019b; Li B. et al., 2020). After controlling for other potential drivers such as culture time and microbial abundance, this correlation still exists. The reason may be that high biodiversity as a biological barrier can resist the spread of antibiotic resistance (Chen et al., 2017b), or microbial communities with high ARGs abundance compete with susceptible species to reduce soil diversity (Van Goethem et al., 2018). Thereby, increasing microbial diversity can slow down the propagation of ARGs in the soil (Chen et al., 2019b).

### Transmission mechanism of antibiotic resistance genes and their relationships with MGEs

**Figure 2:**
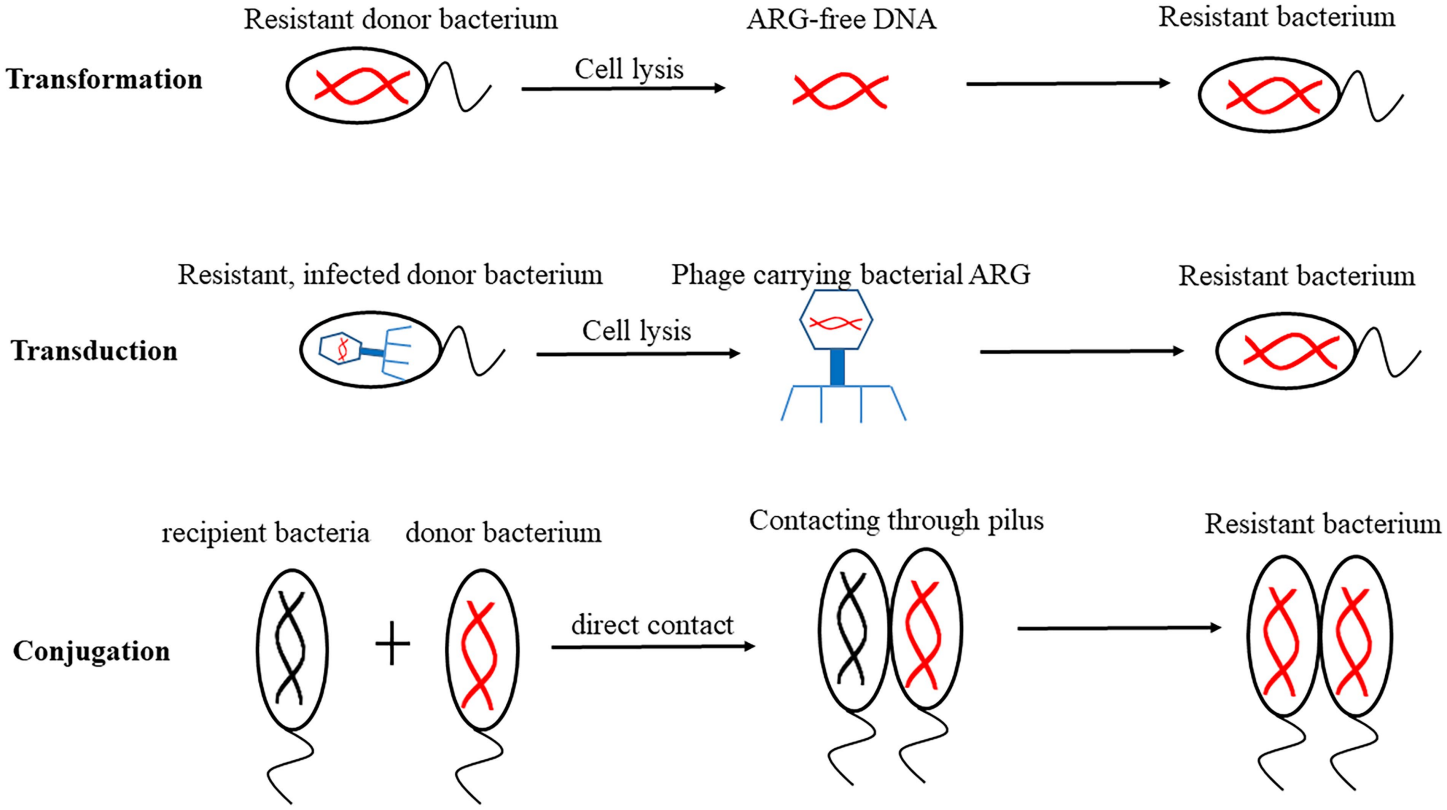
Mechanisms of horizontal gene transfer (HGT). Transformation refers to that the receptive receptor bacterial cells embed the free antibiotic resistance genes (ARGs) into the bacterial chromosome, integrate and stable express. Transduction refers to transfer ARGs from donor bacteria to recipient bacteria by phage. Conjugation refers to transfer of ARGs through sexual pili during direct contact between donor and recipient cells.

ARGs are highly diverse because they can move between microbes (Gorecki et al., 2019). There are two main ways for ARGs to spread in the environment, vertical gene transfer (VGT) and horizontal gene transfer (HGT). VGT refers to that the genomic content passes from generation to generation. Generally, these ARGs are located on the chromosomes. HGT refers to the transfer of MGEs such as plasmids, integrons and transposons carrying ARGs between two strains through transformation, transduction and conjugation, so that the recipient bacteria can obtain new metabolic ability to adapt to the new niche (D’Costa et al., 2007). These ARGs are usually located on plasmids. These three HGT mechanisms are shown in Figure 2. Transformation refers to that the competent receptor bacterial cells embed the free ARGs absorbed from the external environment (ARBs are released into the environment after dissolution) into the bacterial chromosome, then obtain a new phenotype. Transformation is the most common way of HGT in bacteria (Wang, 2019). Transduction refers to the process of transferring ARGs from donor bacteria to recipient bacteria by phage (Barlow, 2009), then conducting gene recombination. Penadés and his colleagues found that phages were active in transmission of ARGs related to MGEs (Penades et al., 2015). Conjugation refers to ARG transfer to recipient cells through sexual pili during direct contact between donor and recipient cells (Abe et al., 2020).

Most MGEs before the antibiotic era did not carry ARGs (Davis and Anandan, 1970; Hughes and Datta, 1983), but with the selective pressure posed by the increased use of antibiotics, more and more MGEs were accompanied with resistance genes. Studies have shown that the total relative abundance of ARGs and the relative abundance of several individual ARGs are significantly positively correlated with total transposase genes and total integrase genes (Pehrsson et al., 2016; Guo et al., 2019; Zhang Y-J. et al., 2019; Li S. et al., 2020). Moreover, we can observe the correlation of antibiotic resistance genes and MGEs after controlling various influencing factors such as pH and temperature. The variation of ARGs can be explained mainly by MGEs, accounting for 36.41%; 10 of 16 ARGs were associated positively with MGEs (Guo et al., 2019). *IntI1* (class I integron-integrase gene) was significantly positively correlated with *sulI*, *sulII* and *tetG* gene in manure-amended soil (Guo et al., 2019). Quinolone resistance genes are prevalent, probably because they are located on plasmids and are easy to transfer between natural bacteria (Vaz-Moreira et al., 2016). In agriculture soils, HGT mediated by MGEs may enable nonpathogenic environmental bacteria and pathogens acquire resistance (Johnson et al., 2016), thus increasing the difficulty of clinical treatment. However, other studies have shown that the association of MGEs and ARGs does not always exist (Zhang et al., 2020), or MGEs cannot explain much about ARG variations (Xu et al., 2021). For example, the absolute abundance of ARGs was significantly correlated with the abundance of integrons, but not with the abundance of transposases in wetland soil on the Qinghai-Tibetan Plateau (Yang et al., 2019). These phenomena need to be studied further.

### The association between antibiotic resistance genes and soil physicochemical properties

The main factors shaping ARGs profiles are distinct in different geographical locations and soil types. Soil pH, moisture, temperature, and C/N ratio all can affect ARGs profiles. Soil pH and moisture are common influencing factors in various soils.

Soil pH is one of the most vital factors affecting ARGs diversity and composition (Xie et al., 2018). ARGs diversity is highest when pH is neutral (Hu H-W. et al., 2018), and positively correlated with pH (pH: 4.0 ~ 5.5) in Amazon rainforest soil (Lemos et al., 2021). Soil pH was significantly and negatively correlated with ARGs abundance in bulk soil (Yan et al., 2021). For instance, the abundance of ARGs (*tetO*, *tetQ*, *tetC* and *tetX*) increased significantly under acidic conditions and decreased under alkaline conditions (Guo A. et al., 2017). Previous studies showed that soil pH is also the essential factor that influences the soil microbial diversity and community structure (Shen et al., 2013; Sun et al., 2015c), and soil pH can select microorganisms by affecting nutrient availability or physiological activity (Xiao et al., 2014). The certain pH levels may exert direct pressure on bacterial cells, selecting some bacterial population (e.g., Acidobacteria; McCaig et al., 1999, Zhang et al., 2014), and then indirectly affect the ARGs profiles. The optimal pH of most soil bacteria is narrow (the intracellular pH value is usually within 1 unit of near neutral pH; Xiao et al., 2016), and the growth of soil microorganisms is inhibited, if pH deviates from the optimal pH (Rousk et al., 2010).

In addition, soil moisture is another key factor driving the ARGs pattern (Hu H-W. et al., 2018; Yang et al., 2019; Zhang et al., 2020). Soil moisture is positively correlated with the relative abundance of ARGs encoding inactivation mechanism (Cheng et al., 2019; Song et al., 2021). The absolute copy numbers of *sulI* and *tetO* gene were positively correlated with moisture (Song et al., 2016), which might be explained that water is one of the main limitations for growth of the total microbial community in soils. Thus increased moisture will increase the microbial biomass, then increase the ARG content. In the thermophilic composting stage, the main factor affecting ARGs is moisture. Low moisture conditions are helpful to removing ARGs (Cheng et al., 2019), because high moisture promotes the propagation of ARGs by MGEs (Wang et al., 2016), or ARGs migrate with water flow. Moreover, the moisture level can also indirectly affect ARGs level by affecting microbial activity (Liang et al., 2003), free airspace (Awasthi et al., 2018), metabolic activity, physiological activity (Zhang M. et al., 2019), ARGs dissipation (Song et al., 2016) and increasing the sensitivity of microbial community to antibiotics (Reichel et al., 2014).

Nutrients in the soil are also correlated with ARGs. For instance, potassium is positively correlated with the relative abundance of ARGs with protective mechanisms or sulfonamides classes (Song et al., 2021). Total phosphorus is negatively correlated with the relative abundance of ARGs encoding efflux pump mechanism or tetracyclines classes (Zhang Y-J. et al., 2019). High C/N ratio and NO_3_^−^-N contents are positively correlated with the absolute abundance of ARGs (Guo et al., 2020). Moreover, the nitrogen conversion indicators (TKN: total Kjeldahl nitrogen, NO_3_^−^-N, NH_4_^+^-N) explain part of the variation of ARGs (24.21%; Guo et al., 2019). The nutrient is one of the most significant determinants for the direction of microbial interaction. In nutrient-rich environment, microorganisms show competition and increases the diversity of ARGs (Hammarlund and Harcombe, 2019).

Soil temperature shapes the distribution of resistome and it is negatively correlated with ARGs abundance. The reason may be that increasing soil temperature decreased microbial community diversity, then leaded to reduce ARGs abundance in soils (Dunivin and Shade, 2018). The diversity and abundance of *bla_A*, *bla_B*, *dfra12* and *tolC* gene decreases with soil temperature (Dunivin and Shade, 2018). High temperature can reduce the abundances of most ARGs (Banerjee et al., 2016), which is usually used to remove ARGs in the composting process.

### The association between antibiotic resistance genes and meteorological parameters

Meteorological parameters (e.g., precipitation and atmospheric temperature) have great effects on ARGs. The abundance, diversity and composition of ARGs increase substantially during rainfall (Jang et al., 2021). Total ARGs suddenly reached to the highest level (4.5 × 10^9^ copies/ml) at the 7th after rainfall (Jang et al., 2021). The average concentrations of *sulII* gene in the wet season (9.04 × 10^7^ copies/g sediments) were higher than those in the dry season (3.78 × 10^7^ copies/g sediments; Li et al., 2018). Moreover, the absolute abundances of the *sul1, sul2, tetA, tetQ, qepA, qnrB, qnrS, ampC,* and *aacC2* gene increased by nearly one order of magnitude during rainfall compared with before rainfall (approximately 12, 16, 3.6, 11, 1.1, 32, 7.5, 5.7, and 3.8 folds, respectively; Wang Q. et al., 2021). Several reasons can explain the phenomena. Firstly, almost 98% of airborne ARB particles fall to the ground surface through the scavenging action of rainwater, promoting the dissemination of ARGs from ambient air to soil during rainfall (Wang Q. et al., 2021). The relative contribution of rain to resistance genes was 16.34% (Hu J. et al., 2018). Secondly, wetter weather can lead to bacterial blooms, compared with drier conditions (Marti et al., 2014). Thirdly, rainwater increased the abundance of MGEs, especially the *intI*1 (2.35 × 10^6^ copies m^−3^), which accelerated the propagation of ARGs (Wang Q. et al., 2021).

It appears that the effect of atmospheric temperature on ARGs was less important than that of precipitation (Meyers et al., 2020). Previously, some studies found that ARGs were positively associated with air temperature. The absolute abundance of resistance genes was highest in summer (2.81 × 10^9^ copies/L on average), and the ARG abundance in four seasons fluctuated along with local air temperature (Zheng et al., 2018). For example, temperature was positively associated with vancomycin and sulfonamide resistance genes (Yang et al., 2019). This may be attributed to the warming climate, which will very likely contribute to increased soil temperature, alter the ARGs-containing microbial community structure and then increase the background levels of ARGs. Climate warming is one of the severe environmental challenges of the world today, and this may contribute to increasing ARGs abundance, especially high risk ARGs. If high-risk ARGs are transferred to human pathogens, this could pose a huge burden for public health. Conversely, the absolute abundances of seven types of ARGs (*tetM*, *tetO*, *tetW*, *ermB*, *ermQ*, *mphE* and *aph (3′)-IIIa*) showed little correlations with temperature (Ouyang et al., 2020). Therefore, the association of ARGs and temperature remains elusive.

### The association between antibiotic resistance genes and antibiotic use

The relationship between antibiotics and ARGs is unclear (Tan et al., 2006; Zhang et al., 2015). Traditionally, according to Darwin’s “arms-shields race” evolutionary hypothesis, antibiotics directly select ARGs. The concentration of antibiotics are positively correlated with the abundance of ARGs (Su et al., 2014; Zhang et al., 2020) and negatively correlated with the diversity of ARGs (Zhao R. et al., 2019). Moreover, high concentrations of antibiotics are associated with class 1 integrons and can accelerate the spread of ARGs (Andersson and Hughes, 2014).

However, the effect of antibiotics on ARGs is not always strong, sometimes weaker than other drivers, such as MGEs and metals. The reason may be that antibiotics can inactivate rapidly through adsorption, photolysis and biodegradation, so the effect of antibiotics on ARGs profiles may be temporal. The effect of antibiotic concentration on ARGs abundance is insignificant in paddy soil (Zhao et al., 2020). Moreover, antibiotics and their corresponding ARGs do not always appear synchronously, and ARGs can persist even without antibiotic selection pressure. In a remote Alaskan soil that was not contaminated by antibiotics, *bla_LRA-13_* gene (β-Lactamase resistance genes) was detected (Allen et al., 2009). The direct selection pressure of given antibiotics leads to the enrichment of corresponding and non-corresponding ARGs, which reveals the collateral effect of antibiotics on the development of resistance. Although sulfonamides were not detected in soils, *sul* gene (sulfonamides resistance genes) with high abundance was detected in all soil samples (Wang et al., 2015).

### The association between antibiotic resistance genes and heavy metals

The content of heavy metals in soils is generally positively correlated with ARGs, but the soil type should be considered. Cu is positively correlated with aminoglycosides (*aadA* and *aac*) and MLSB (*mefA*) resistance genes detected in Belfast, Northern Ireland soil (Zhao Y. et al., 2019). Zn, Cu and Cd are positively associated with vancomycin resistance genes in the soil of gold tails (Qiao et al., 2021). The strong correlation between heavy metals and ARGs implicates that heavy metals (not easy to degrade) impose continuous selective pressure on metal resistance genes (MRGs). ARGs and MRGs may be located in the same DNA fragment (Kiran et al., 2015). Class I integrons (*intI*) generally exist in metal-polluted environment (Poole, 2017). Heavy metals can combine antibiotics to promote the transmission of ARGs through co-selection by *intI* (Seiler and Berendonk, 2012). For example, Cd/Zn resistance gene (*cadD*) and aminoglycoside resistance gene (*aph (3′) IIIA*) are located on the same plasmid, and β-Lactam resistance gene (*blaCTX-M*) and Cu resistance gene (*pcoA-E (5/25)*) are on the same *IncHI2* plasmid (Fang et al., 2016). Therefore, the transmission of corresponding resistance genes can be accelerated in metal-polluted environment. In turn, microbes may utilize similar mechanisms to fight antibiotics and heavy metals. Furthermore, to some extent, the use of metal-containing antimicrobial agents may promote the occurrence of multidrug resistance (Pal et al., 2017). However, heavy metals do not always correlate with ARGs depending on the concentration of heavy metals. Low heavy metal concentration has little or no effect on ARG profiles; On the contrary, high heavy metal concentration greatly impacts ARG profiles (Wang X. et al., 2021). Low concentration of Ni (the geo-accumulation index (Igeo) < 0 in some samples) is not related to most of the ARGs detected; High concentrations of As, Pb and Cd (Igeo>4 in some samples) are positively correlated with aminoglycoside and vancomycin resistance genes (Qiao et al., 2021).

### The association between antibiotic resistance genes and other pollutants

The PAHs in soils, including pyrene, benzopyrene, phenanthrene and naphthalene, can affect the pattern of ARGs. The expression of ARGs cassette elevated in PAHs-contaminated soil, and the fluctuation of the abundance of tetracycline resistance genes (*tetM*, *tetW*) and sulfonamide resistance genes (*sulII*, *sulIII*) was positively correlated with the pyrene concentration in soil (Sun et al., 2015a). The 100 mg L^−1^ naphthalene and 10 mg L^−1^ phenanthrene increased the abundances of sulfonamide resistance gene (*sulI*) and aminoglycoside resistance gene (*aadA2*; Wang et al., 2017). This is possibly because PAH directly enriches ARGs and produces mutagenic effect by triggering stress/repair system or changing DNA composition (Busch et al., 2018). In addition, the abundances of ARGs such as *macB*, *mexB* and *tolC* in PAHs-contaminated soil were about 15 times higher than those in lightly polluted soil. ARGs enriched in PAHs-contaminated soil are mostly in chromosomes rather than plasmids, so their frequencies of HGT among bacteria are low (Chen et al., 2017a). Phenanthrene, a small molecule, leaded to the reduction of ampicillin resistance gene (*Ampr*) transformation through noncovalent interaction; the transformation was not significant in the presence of macromolecule pyrene and benzopyrene, which further indicated the reduction of HGT in PAH-contaminated samples (Kang et al., 2015). However, the total amount of phenanthrene or pyrene in soil was not always related to ARGs abundance, but their bioavailability was significantly related to ARGs abundance (Sun et al., 2015b), thus, the relationship between PAHs and ARGs needs further study.

Pesticides and antibiotics together exert selective pressure on microorganisms to produce pesticide-antibiotic cross-resistance (Rangasamy et al., 2018). The application of pesticides increased the absolute and relative abundance of *ermB*, *aph (3′)-IIIa* and *tetW* gene in the soil quickly (Radu et al., 2021). The 20.0 mg kg^−1^ chloropyrophos significantly increased the total abundance of *tetM*, *tetO*, *tetQ*, *tetW*, *tetX*, *sulI* and *sulII* gene (Guo et al., 2020). Furthermore, bacteria in pesticide-contaminated soil contained *IncP* plasmid, which further promoted the propagate of ARGs (Anjum et al., 2011).

### The association between antibiotic resistance genes and plants

Plants can acquire exogenous ARGs by soil. The dynamics and transfer of ARGs amongst plants are associated with soil. On one hand, plant phyllosphere and rhizosphere bacteria can absorb exogenous ARGs from soil (Chen et al., 2019a). Ten ARGs (*vanTC*, *vanC*, *vanYD*, *mexF*, *ttgA*, *oprJ*, *ampC*, *blaCTX-M*, *pncA*, *cmx (A)*) were absorbed into Brassica phyllosphere from soil (Chen et al., 2017c). On the other hand, soil ARGs can be horizontally transferred to microbiomes that parasitize or adhere to plants (Chen et al., 2019c). Moreover, the *intI1* and genes encoding transposases are common in vegetables (Wang et al., 2015; Chen et al., 2018). Some self-transmissible plasmids resisting tetracycline occur in arugula (Blau et al., 2018), which speeds up transferring ARGs from soil to plants. High risk ARGs in vegetables may be transferred to the human gut and may harm human health if ARGs reside in the body.

## The association between antibiotic resistance genes and human health

The relationship between antibiotic resistance and human health can be characterized by the soil–plant/animal-human cycle in soil. Antibiotics, ARGs or ARBs are brought into the soil by wastewater inflow, reclaimed water irrigation, composting and other ways. Then, plants can absorb ARGs from soil. Fortunately, most plant ARG-carrying microbes are non-pathogenic (Zhang et al., 2011), but their possible participating in the spread of ARGs to human pathogens by HGT *via* MGEs (Rossi et al., 2014). Endophytic bacteria of plants closely related to human pathogens or opportunistic human pathogens may potentially harm human health. In 2011, multidrug resistant enterohemorrhagic *Escherichia coli* (EHEC) broke out in Europe, causing 50 persons dead by eating raw fruits and vegetables contaminated by animal feces (Buchholz et al., 2011). Outbreaks of *Salmonella poona* infections in America associated with consuming melons were associated to unhygienic irrigation at the source farms (Lee and Gilmore, 2004). Therefore, ARGs in soil spread to vegetables and threaten human health if vegetables are not or rarely processed.

Animals intake ARGs from soil through contact, feedings, and other ways, acting as intermediate hosts for ARBs and becoming reservoirs for ARGs to transfer to human pathogens (Allen et al., 2010). Then, humans acquire ARGs by eating animal products (Winokur et al., 2001), which causes direct and indirect harm to human health. Mcr-1 myxin resistance genes were initially found in animals and meat and then detected in food samples and human intestinal flora (Hu et al., 2016), indicating that ARGs were transmitted from animals to humans. It is reported that the outbreak of quinolone-resistant *Campylobacter* infections in the United States is caused by humans consumption of chicken (Barza and Travers, 2002).

Antibiotics are one of the greatest discoveries before the Second World War. The mortality rate of infectious diseases drops sharply by antibiotics treatment (Rachakonda and Cartee, 2004). Therefore, the liberal use of antibiotics in clinical practice causes widely-distributed antibiotic resistance, which leads infectious diseases to be one of the leading causes of death in the world (Nathan, 2004). In Europe, the *Escherichia coli* resistance to the third generation cephalosporins increases (European Center for disease prevention and control, 2016). Cephalosporins, as a common over-the-counter drug in China, its resistance consequences have certain reference implications.

Moreover, the excessive use and abuse of various antibiotics by humans has led to the emergence of multidrug-resistant bacteria in the environment and human body, and even “superbacteria” such as NDM-1 (Ahammad et al., 2014; Nesme and Simonet, 2015). Then, the pathogens has become insensitive to most antibiotics used in the clinic (World Bank, 2017). Previously, highly pathogenic *Klebsiella pneumonia* was detected in Chinese hospitals, resistant to all tested antibiotics (Gu et al., 2018). These make us stand on the edge of the post-antibiotic era (Tyrrell et al., 2019). The American Centers for Disease Control and Prevention estimates that more than 70% of the bacteria causing the infection are resistant to at least one antibiotic commonly used for treatment (IDSA, 2004). This has greatly increased the treatment difficulty of clinical infectious diseases and increased infection mortality. By 2050, it is expected that 10 million people will die of antibiotic resistance every year (WHO, 2019), which will bring a substantial economic burden to patients and society. A WHO report point out that by 2050, the global financial burden caused by antibiotic resistance will be equivalent to that caused by the economic crisis in 2008 (WHO, 2019).

## Future perspectives

Since the discovery of ARGs in the last century, people constantly get the knowledge about it, from what to how to spread and then to harm. However, much remains unknown. Firstly, which technologies can reduce the source and presence of ARGs in soil? Secondly, what are the molecular mechanisms of environmental factors affecting soil ARGs transmission? Thirdly, how does soil ARGs affect human health, and how can it be mitigated? Some work needs to be done to figure out these problems. The following initiatives are put forward for future research:

1. Develop highly efficient technologies to treat agricultural waste, waste water and minimize their ARGs input to soil. Materials introduced into soil, such as ARGs in compost and irrigation water, should be treated in advance to reduce input. Establish a sound surveillance system to reduce the release of ARG into the soil.
2. Investigate the influence mechanisms of special substrates in soil, such as clay minerals and humic acid, on the genetic transformation and horizontal gene transfer of ARGs; The effects of different ARGs in soil environment and their harm to human body may vary greatly, therefore, it is necessary to analyze the environmental and health effects of important ARGs in soil from the perspective of molecular mechanism.
3. Continue to deeply study the transfer and transformation regulation of ARGs in soil, clarify the individual and compound effects of various environmental and biological factors, establish biogeochemical models, predict the migration and fate of ARGs in soil, and provide scientific guidance for biological governance.
4. Develop strategies to regulate soil ARGs. Whether ARGs can be modified to reduce the impact on the environment and human health through cultivation, fertilization, irrigation and other measures.
5. Formulate reasonable and standardized antibiotic use measures and legal norms to reduce the abuse of antibiotics, especially in agriculture, so as to reduce ARGs in soil environment.

## Conclusion

ARGs are indigenous and ancient in soil, and soil can acquire exogenous ARGs from various ways. Microbiome, as the producer and potential host of ARGs, its composition and structure shape ARGs profiles, which acquire resistance by HGT *via* MGEs. ARG abundance are negatively correlated with pH, but are positively associated with moisture and rainfall amount. Antibiotics, heavy metals, PAHs and pesticides in high concentration can exert selective pressure on ARGs and enrich ARGs. Plants and animals can absorb ARGs from soil, then spread to humans and possibly pose a potential harm to human health. Our study systematically reviewed various influencing factors of soil resistome, such as microorganisms, MGEs. Although we have acquired these achievements of ARGs, there are still many unknowns that need to be further studied to protect humanity from ARGs. In the future, we need to study how soil ARGs are transmitted to the human body and affect human health and how this potential harm can be reduced.

## Author contributions

BH, LM, SZ, and HL led the drafting of the manuscript, with substantial input from the other authors. All authors contributed to the article and approved the submitted version.

## Funding

This work was supported by the National Natural Science Foundation of China (42007026), NSRP of CQTGMC (XJ2021000101), and Innovative Research Group Project of Chongqing municipal education commission (CXQT20030).

## Conflict of interest

The authors declare that the research was conducted in the absence of any commercial or financial relationships that could be construed as a potential conflict of interest.

## Publisher’s note

All claims expressed in this article are solely those of the authors and do not necessarily represent those of their affiliated organizations, or those of the publisher, the editors and the reviewers. Any product that may be evaluated in this article, or claim that may be made by its manufacturer, is not guaranteed or endorsed by the publisher.
